# Publisher Correction: Cryo-EM structure of cortical microtubules from human parasite *Toxoplasma gondii* identifies their microtubule inner proteins

**DOI:** 10.1038/s41467-021-24385-1

**Published:** 2021-06-25

**Authors:** Xiangli Wang, Yong Fu, Wandy L. Beatty, Meisheng Ma, Alan Brown, L. David Sibley, Rui Zhang

**Affiliations:** 1 Department of Biochemistry and Molecular Biophysics, Washington University in St. Louis, School of Medicine, St. Louis, MO USA; 2 Department of Molecular Microbiology, Washington University in St. Louis, School of Medicine, St. Louis, MO USA; 3 Department of Biological Chemistry and Molecular Pharmacology, Blavatnik Institute, Harvard Medical School, Boston, MA USA

**Keywords:** Microtubules, Parasite biology, Cryoelectron microscopy

Correction to: *Nature Communications* 10.1038/s41467-021-23351-1, published online 24 May 2021.

The original version of this Article contained an error in the spelling of the author L. David Sibley, which was incorrectly given as David L. Sibley. This has now been corrected in both the PDF and HTML versions of the Article.

