# Supplementary material for: Cryo-EM structure of cortical microtubules from human parasite Toxoplasma gondii identifies their microtubule inner proteins
Source: Nat Commun. 2021 May 24;12:3065. doi: 10.1038/s41467-021-23351-1 (PMC8144581; doi:10.1038/s41467-021-23351-1)
Supplement: Supplementary file 1 — Supplementary Information [file 41467_2021_23351_MOESM1_ESM.pdf]

Supplementary Information

Cryo-EM structure of cortical microtubules from human parasite Toxoplasma gondii identifies their microtubule inner proteins

Xiangli Wang, Yong Fu, Wandy L. Beatty, Meisheng Ma, Alan Brown, L. David Sibley, Rui Zhang

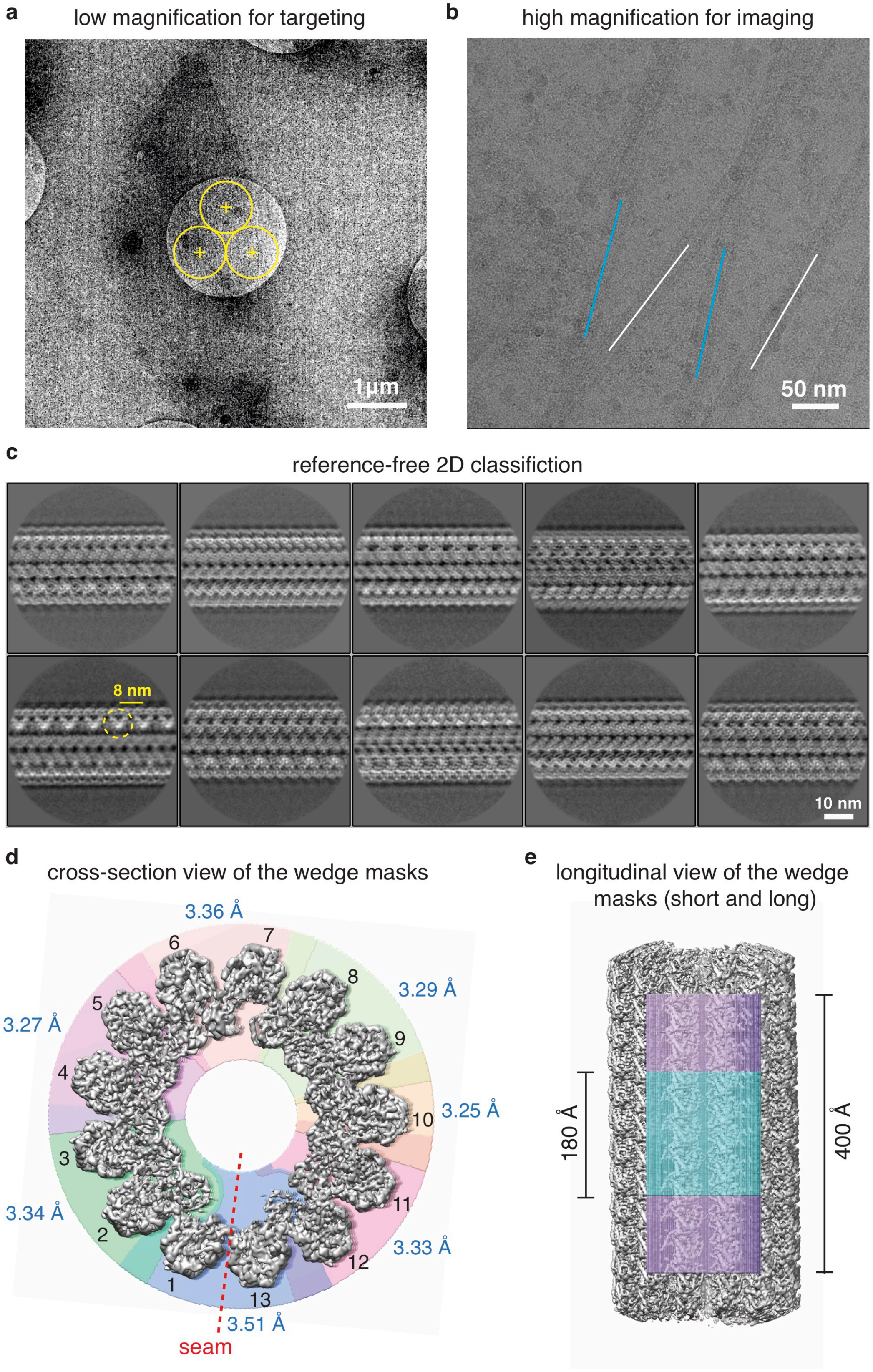

a
low magnification for targeting
1µm
b
high magnification for imaging
50 nm
c
reference-free 2D classifiction
8 nm
10 nm
d
cross-section view of the wedge masks
3.36 Å
3.29 Å
3.27 Å
3.25 Å
3.34 Å
3.33 Å
3.51 Å
1
2
3
4
5
6
7
8
9
10
11
12
13
seam
e
longitudinal view of the wedge masks (short and long)
180 Å
400 Å

Supplementary Fig. 1 | Cryo-EM data collection and data processing. a, One representative low magnification image (out of a total of 5,160 images) of the cryo-EM grid used by EPU software to set up three exposure targets per hole (2 μm in diameter). The size of the electron beam is indicated by the yellow circles. **b**, A typical motion-corrected cryo-EM micrograph (out of a total of 9,231 movies) of our sample. Two sets of parallel MT arrays (white and blue) can be observed in this micrograph, corresponding to the two sets of cortical MTs located at opposite sides of the cell body (see Fig. 1a, solid versus dashed lines). **c**, Result of reference-free 2D classification of the particle images generated by Relion-3. Class averages of ten classes (out of 100 classes) with the most number of particles, showing the presence of MIP densities with 8-nm periodicity. **d**, To improve local map quality, we divided the structure into seven sub-regions using soft-edged wedge masks, each covering 2 adjacent protofilaments (P2-3, P4-5, P6-7, P8-9, P10-11, P11-12, P13-1). The shapes of the wedge mask were modified for P11-P12, P13-1 and P2-3 to preserve the best quality of TrxL1/2 densities. The resolution numbers (reported by cryoSPARC) for different sub-regions are provided. **e**, For each sub-region, a wedge mask with longer length (400 Å) was used for 3D classification (*Class3D* in Relion-3), while a wedge mask with shorter length (180 Å) was used for local refinement (*Refine3D* in Relion-3 or *local refinement* in cryoSPARC).

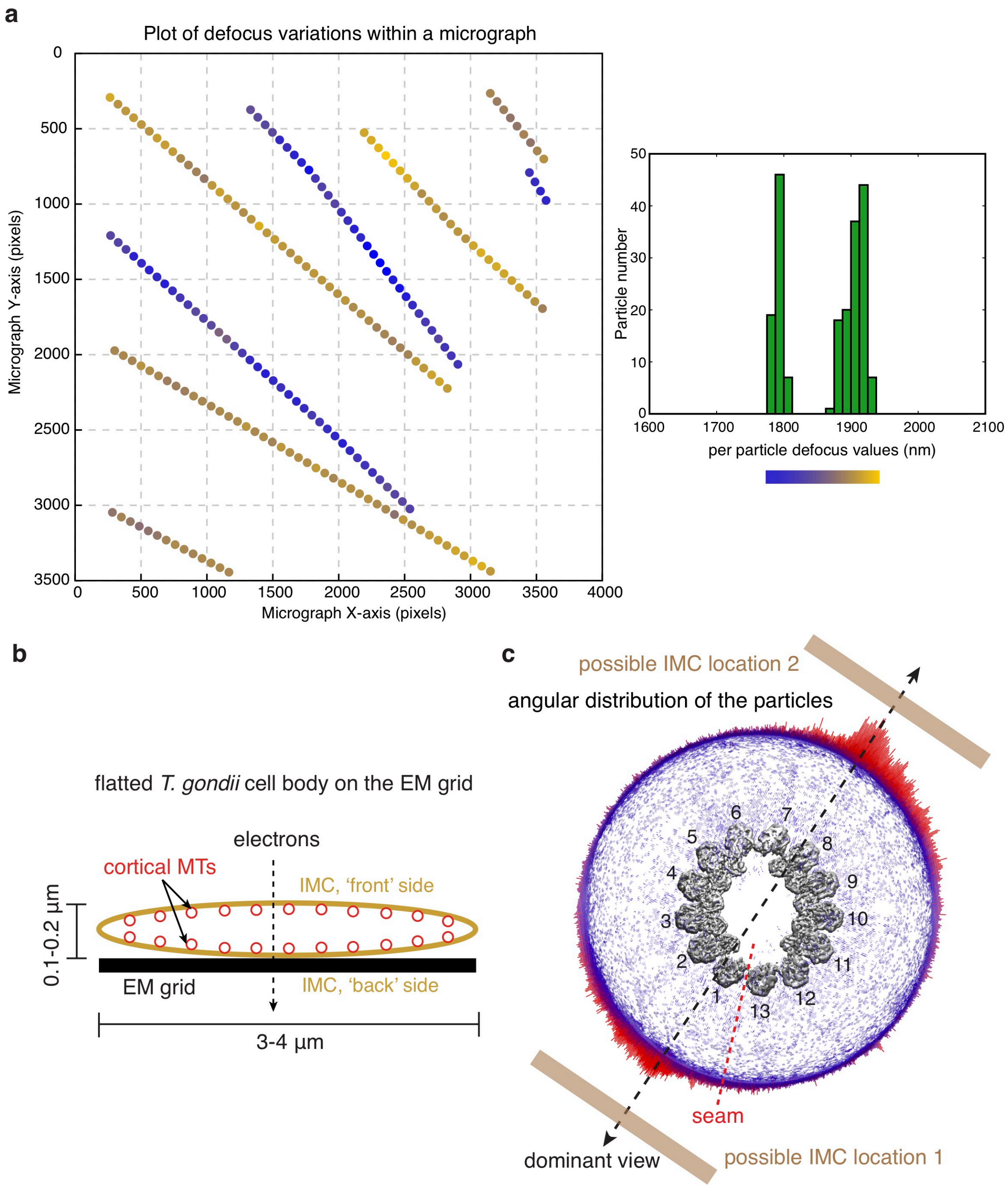

Supplementary Fig. 2 | The parasites are significantly flattened on the EM grid. a, Left: plot of defocus variations within a cryo-EM micrograph. Each dotted line corresponds to an individual MT (each dot is a MT particle). Right: histogram of number of particles versus their

defocus values. From dark blue color to yellow, the per-particle defocus values change from -1.777 μm to -1.931 μm (154 nm difference). There are clearly two sets of defocus values that correspond to the two sets of cortical MTs located at the 'front' and 'back' sides of the IMC. **b**, Schematic of a significantly flattened cell body of *T. gondii* on the EM grid. The width of the cell bodies on the EM grid was estimated to be 3-4 μm based on the low magnification images of the cryo-EM grid as shown in Supplementary Fig. 1a, while the thickness between the front and back surfaces of the flattened cell bodies was estimated to be 0.1-0.2 μm using the plots of defocus variations as shown in panel a. **c**, Plot of angular distribution of all the MT particles revealed a dominant view. The heights of bars (color coded from blue to red) along each direction is proportional to the number of particles assigned with the corresponding angular orientation. Two possible locations and orientations of the IMC are indicated, as single particle EM cannot distinguish the front and back surfaces of the IMC. The IMC is drawn approximately 23 nm apart from the cortical MT based on a previous cryo-ET study[6]. Both plots in a and c were generated by Relion-3 software[57]. The anisotropic angular distribution does not seem to affect the quality of the 3D reconstruction, probably due to the fact that we have a large dataset and therefore sufficient number of particles for the non-dominant views.

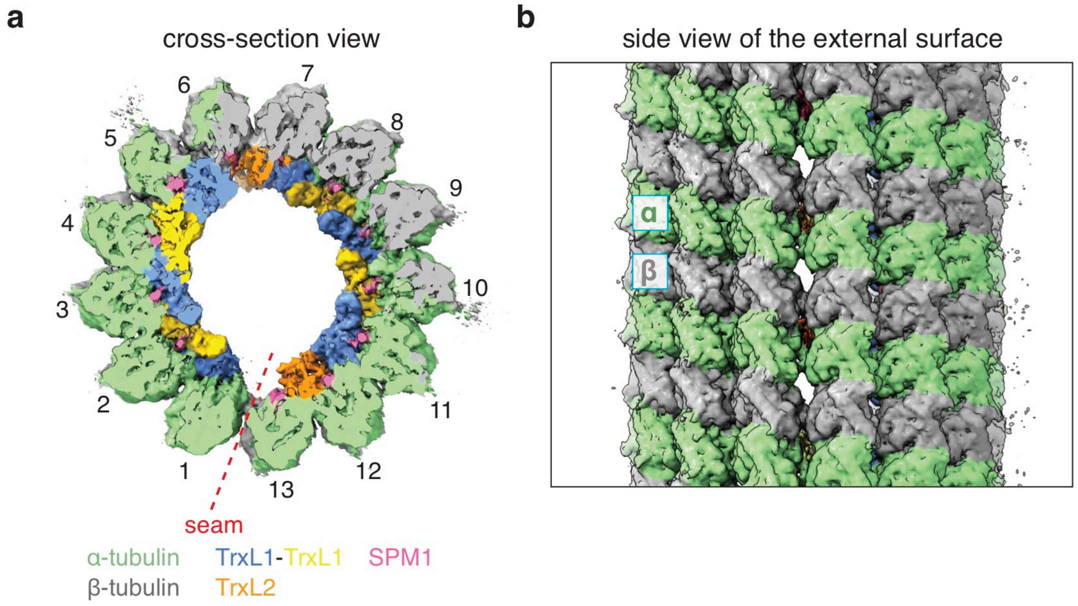

Supplementary Fig. 3 | Cryo-EM structure of *T. gondii* cortical MT lacks external MAPs. a, Cross-section view of the unsharpened cryo-EM density map displayed at low isosurface threshold without a mask. Only weak disordered densities can be seen near protofilaments P5, P9 and P10, which should correspond to the tubulin C-terminal tails. **b**, Same as a, but showing the longitudinal view of the external surface.

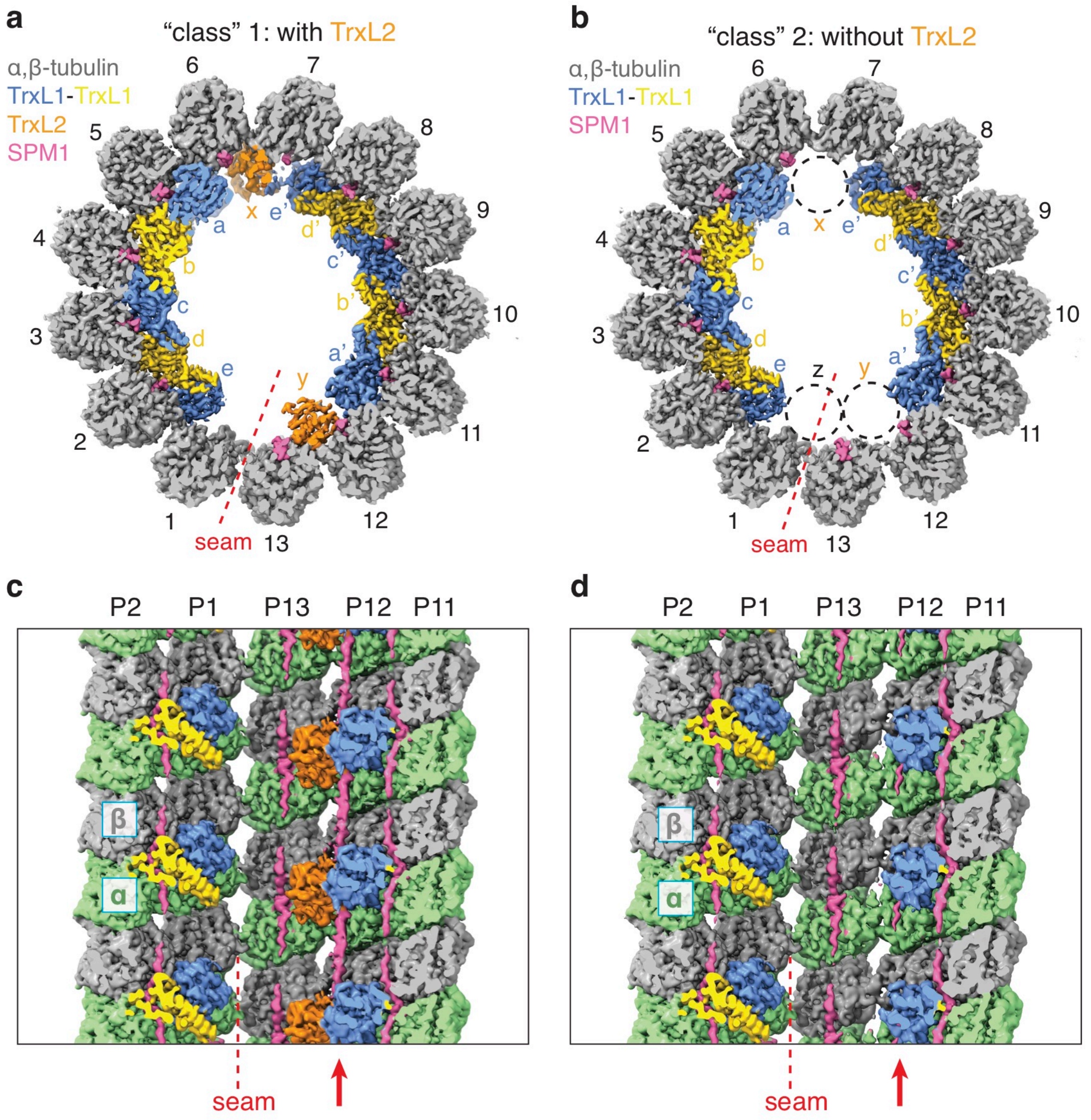

Supplementary Fig. 4 | The arrangement of MIPs on protofilaments across the MT seam. a-b, Identical to Fig. 1b,c. Cross section view of the cryo-EM structure of *T. gondii* cortical MT with (a) and without (b) TrxL2 at sites x and y. **c-d**, Luminal view of the MIPs on protofilaments across the MT seam. Unsharpened composite maps with (c) and without (d) TrxL2 (see Methods) were used for visualization. Note in the presence of TrxL2, the SPM1 density forms a continuous linear density on protofilament P12 (red arrow); while in the absence of TrxL2, the SPM1 density on P12 is much weaker (red arrow).

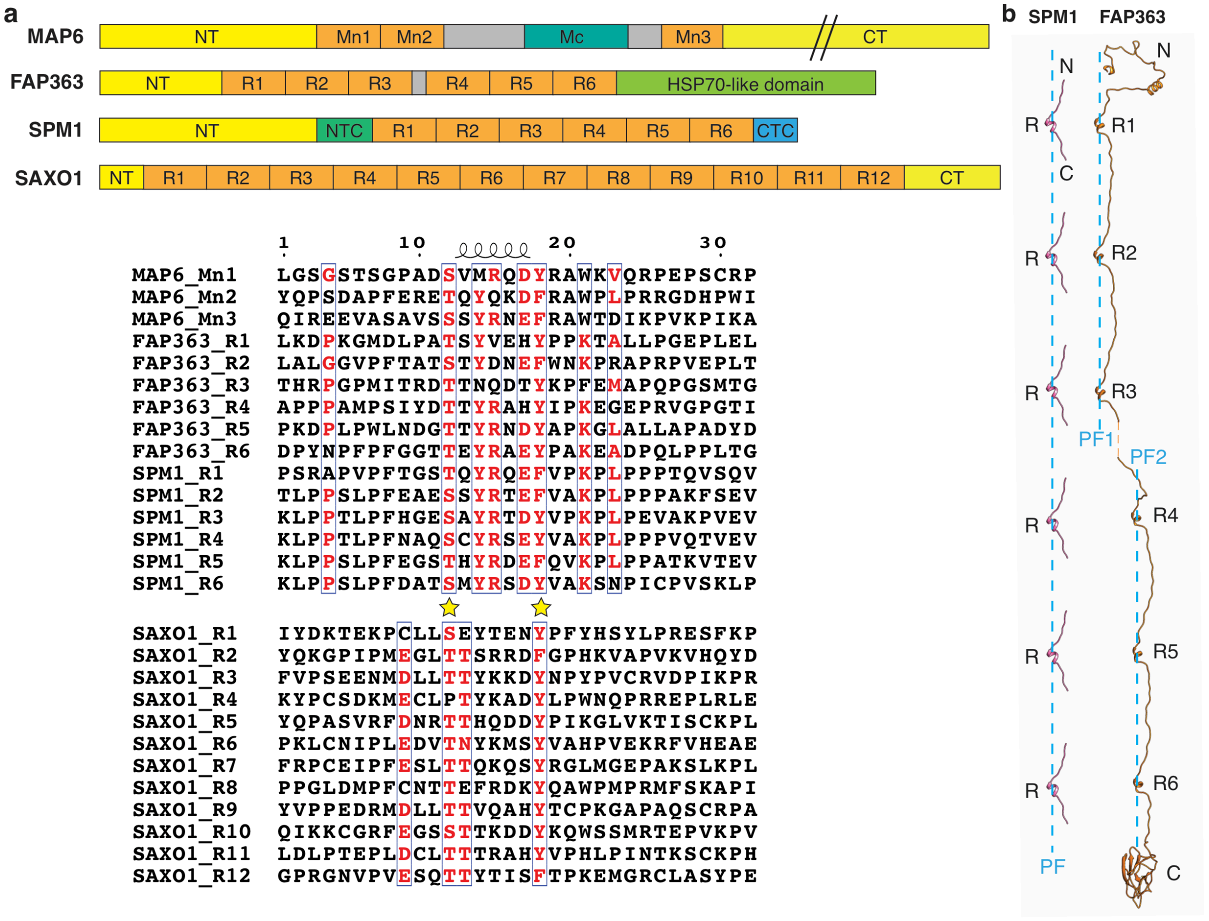

Supplementary Fig. 5 | The consensus Mn motif shared between SPM1 and other proteins. **a**, Above: schematic of the domain organization of human MAP6, *Chlamydomonas* FAP363, *T. gondii* SPM1 and human SAXO1 proteins. NT = N-terminal domain; CT = C-terminal domain; NTC = N-terminal conserved domain (among Apicomplexa); CTC = C-terminal conserved domain (among Apicomplexa). Grey boxes are spacers between MT stabilizing motifs. Below: Sequence alignment of the Mn motifs from these proteins featuring two conserved T/S and F/Y residues (yellow stars) that mark the start and end positions of a short helix (indicated above the sequences) that bind at the interface between α and β-tubulin, as shown in Fig. 3d. **b**, Atomic models of FAP363 and SPM1 on MTs. Each blue dashed line indicates the axis of one protofilament. Note FAP363 spans two adjacent protofilaments.

```
Pig_TUBA1B           β1  α1  TT  η1  β2  β3
                     1         10         20         30         40         50         60
Pig_TUBA1B           .MRECISIHVGQAGVQIGNACWELYCLEHGIQPDGQMPSDK...TIGGGDDSFNTFFSETGAGKHVPRAV
Human_TUBA1A         .MRECISIHVGQAGVQIGNACWELYCLEHGIQPDGQMPSDK...TIGGGDDSFNTFFSETGAGKHVPRAV
Chlamy_TUBA1         .MREVISIHIGQAGIQVGNACWELYCLEHGIQPDGQMPSDK...TIGGGDDAFNTFFSETGAGKHVPRCI
Tox_583.m00022_α1    .MREVISIHVGQAGIQIGNACWELFCLEHGIQPDGQMPSDK...TIGGGDDAFNTFFSETGAGKHVPRCV
Tox_44.m02671_α2     .MREIISLHIGQAGVQVGNACWELFCLEHGIGSGGRRVDQVNGGDFISSDSSFNAFFAETAQKQYVPRCV
Tox_44.m02659_α3     MPGEVVTVQCGQAGLQLGFAFWELLLAEHGLTYEGTQGKHN...APEGCQNNVDCFFYEAHTGRSVPRSA

Pig_TUBA1B           β4  α2  η2  β5  α3  η3  α4
                     70         80         90         100        110        120        130
Pig_TUBA1B           FVDLEPTVIDEVRTGTYRQLFHPEQLITGKEDAANNYARGHYTIGKEIIDLVLDRIRKLADQCTGLQGFL
Human_TUBA1A         FVDLEPTVIDEVRTGTYRQLFHPEQLITGKEDAANNYARGHYTIGKEIIDLVLDRIRKLADQCTGLQGFL
Chlamy_TUBA1         FLDLEPTVVDEVRTGTYRQLFHPEQLISGKEDAANNFARGHYTIGKEIVDLALDRIRKLADNCTGLQGFL
Tox_583.m00022_α1    FLDLEPTVVDEVRTGTYRHLFHPEQLISGKEDAANNFARGHYTIGKEIVDLSLDRIRKLADNCTGLQGFL
Tox_44.m02671_α2     MVDLEPTVVDDVRKGSYGKLFHPDYLLSGKEDAANNFARGYYTVGREMADTVMECIRRLADNCDGLQGFL
Tox_44.m02659_α3     MIDLDNSAESLTGWSPIRRLFDDEFSVSHKEDASSNFARGYISVGPSVLSAANDVIRRQAEMCDSLQSFF

Pig_TUBA1B           β6  α5  β7  α6  β8
                     140        150        160        170        180        190        200
Pig_TUBA1B           VFHSFGGGTGSGFTSLLMERLSVDYGKKSKLEFSIYPAPQVSTAVVEPYNSILTTHTTLEHSDCAFMVDN
Human_TUBA1A         VFHSFGGGTGSGFTSLLMERLSVDYGKKSKLEFSIYPAPQVSTAVVEPYNSILTTHTTLEHSDCAFMVDN
Chlamy_TUBA1         VFNAVGGGTGSGLGSLLLERLSVDYGKKSKLGFTVYPSPQVSTAVVEPYNSVLSTHSLLEHTDVAVMLDN
Tox_583.m00022_α1    MFNAVGGGTGSGLGCLLLERLSVDYGKKSKLNFCSWPSPQVSTAVVEPYNSVLSTHSLLEHTDVAVMLDN
Tox_44.m02671_α2     VFHAVGGGTGSGLGCHLMETLSVEFARKPKLNFCVWPSPQVSTSVVEPYNAVLSSHSLLEYTDVAVILDN
Tox_44.m02659_α3     VCRSLGGGTGSGLGSLLLEGIAEQFGKKYILDSFVWPFNSQTSSVVEPYNAVLATPTICAHSSAALLVDN

Pig_TUBA1B           α7  α8  α9  TT  β9
                     210        220        230        240        250        260        270
Pig_TUBA1B           EAIYDICRRNLDIERPTYTNLNRLISQIVSSITASLRFDGALNVDLTEFQTNLVPYPRIHFPLATYAPVI
Human_TUBA1A         EAIYDICRRNLDIERPTYTNLNRLIGQIVSSITASLRFDGALNVDLTEFQTNLVPYPRIHFPLATYAPVI
Chlamy_TUBA1         EAIYDICRRSLDIERPTYTNLNRLIAQVISSLTASLRFDGALNVDITEFQTNLVPYPRIHFMLSSYAPII
Tox_583.m00022_α1    EAIYDICRRNLDIERPTYTNLNRLIAQVISSLTASLRFDGALNVDVTEFQTNLVPYPRIHFMLSSYAPII
Tox_44.m02671_α2     EAVYDICRRNLDIQIPSYCDLNRIIAQVISSVTTSLRFDGALNVDMTEYQTNLVPYPRIHFLLTSYAPLL
Tox_44.m02659_α3     AALFNISRNLLNVESVFHSNLNHLVAQVLSATTLSTRFEGTLNSTIEHTLVNTIPYPELKFLTASLAPLT

Pig_TUBA1B           α10  α11  η4  η5  β10  α12  TT
                     280        290        300        310        320        330        340
Pig_TUBA1B           SAEKAYHEQLSVAEITNACFEPANQMVKCDPRHGKYMACCLLYRGDVVPKDVNAAIATIKTKRSIQFVDW
Human_TUBA1A         SAEKAYHEQLSVAEITNACFEPANQMVKCDPRHGKYMACCLLYRGDVVPKDVNAAIATIKTKRTIQFVDW
Chlamy_TUBA1         SAEKAYHEQLSVAEITNAAFEPASMMVKCDPRHGKYMACCLMYRGDVVPKDVNASVATIKTKRTIQFVDW
Tox_583.m00022_α1    SAEKAYHEQLSVAEITNSAFEPASMMAKCDPRHGKYMACCLMYRGDVVPKDVNAAVATIKTKRTIQFVDW
Tox_44.m02671_α2     SASKYRHEQMNVREITVAAFEERNMMAKCCPSKGKYMACCLMYRGDVTPKDVNAAMGTIKSRRDINFVDW
Tox_44.m02659_α3     NLKKYQFERASTKDITIECVLPSRCFASIDYTKNRSIACHIMYRGDVTPRDVQEGVAAVKEHGTLRFVDW

Pig_TUBA1B           β11  TT  β12  η6  α13  α14
                     350        360        370        380        390        400        410
Pig_TUBA1B           CPTGFKVGINYQPPTVVPGGDLAKVQRAVCMLSNTTAIAEAWARLDHKFDLMYAKRAFVHWYVGEGMEEG
Human_TUBA1A         CPTGFKVGINYQPPTVVPGGDLAKVQRAVCMLSNTTAIAEAWARLDHKFDLMYAKRAFVHWYVGEGMEEG
Chlamy_TUBA1         CPTGFKCGINYQPPTVVPGGDLAKVQRAVCMISNSTAIGEIFSRLDHKFDLMYAKRAFVHWYVGEGMEEG
Tox_583.m00022_α1    CPTGFKCGINYQPPTVVPGGDLAKVMRAVCMISNSTAIAEVFSRMDHKFDLMYAKRAFVHWYVGEGMEEG
Tox_44.m02671_α2     CPTGFKTGLNYQPPTFVPDSHLAAAPRSVCMMSNSTAIKEVIQRIDIKFDKMYSKRAFVHWFVGEGMEEG
Tox_44.m02659_α3     IPTGLKCSMNSRLMCVSPESELAPAFQSCCMLANNTGITSLLEKTLADMYKMFNKRAFVHWFVGEGLEEG

Pig_TUBA1B           α15
                     420        430        440        450
Pig_TUBA1B           EFSEAREDMAALEKDYEEVGVDSV.....EGEGEEEG..EEY............................
Human_TUBA1A         EFSEAREDMAALEKDYEEVGVDSV.....EGEGEEEG..EEY............................
Chlamy_TUBA1         EFSEAREDLAALEKDFEEVGAESA.....EGAGEGEG..EEY............................
Tox_583.m00022_α1    EFSEAREDLAALEKDYEEVGIETA.....EGEGEEEGYGDEY............................
Tox_44.m02671_α2     QLTEARDDLAALERDYDEVASDTK.....MDADDEEDLNEDFFPN.........................
Tox_44.m02659_α3     EFLEASERLQRTIDDYHESLIDCEYYYEYEDEGHEGGQGKETAPSESTTEPSSPVHGKREGEEKEPGGTS

Pig_TUBA1B

Pig_TUBA1B           ..
Human_TUBA1A         ..
Chlamy_TUBA1         ..
Tox_583.m00022_α1    ..
Tox_44.m02671_α2     ..
Tox_44.m02659_α3     EV
```

Supplementary Fig. 6 | Sequence alignment of α-tubulin from *T. gondii* and other species. Sequence alignment of α-tubulin from pig, human, *Chlamydomonas* and *T. gondii* (isoforms α1, α2 and α3). The figure was prepared using ESPript[69] based on alignment results from Clustal Omega[70]. Secondary structure elements of α-tubulin from pig are shown above the sequences.

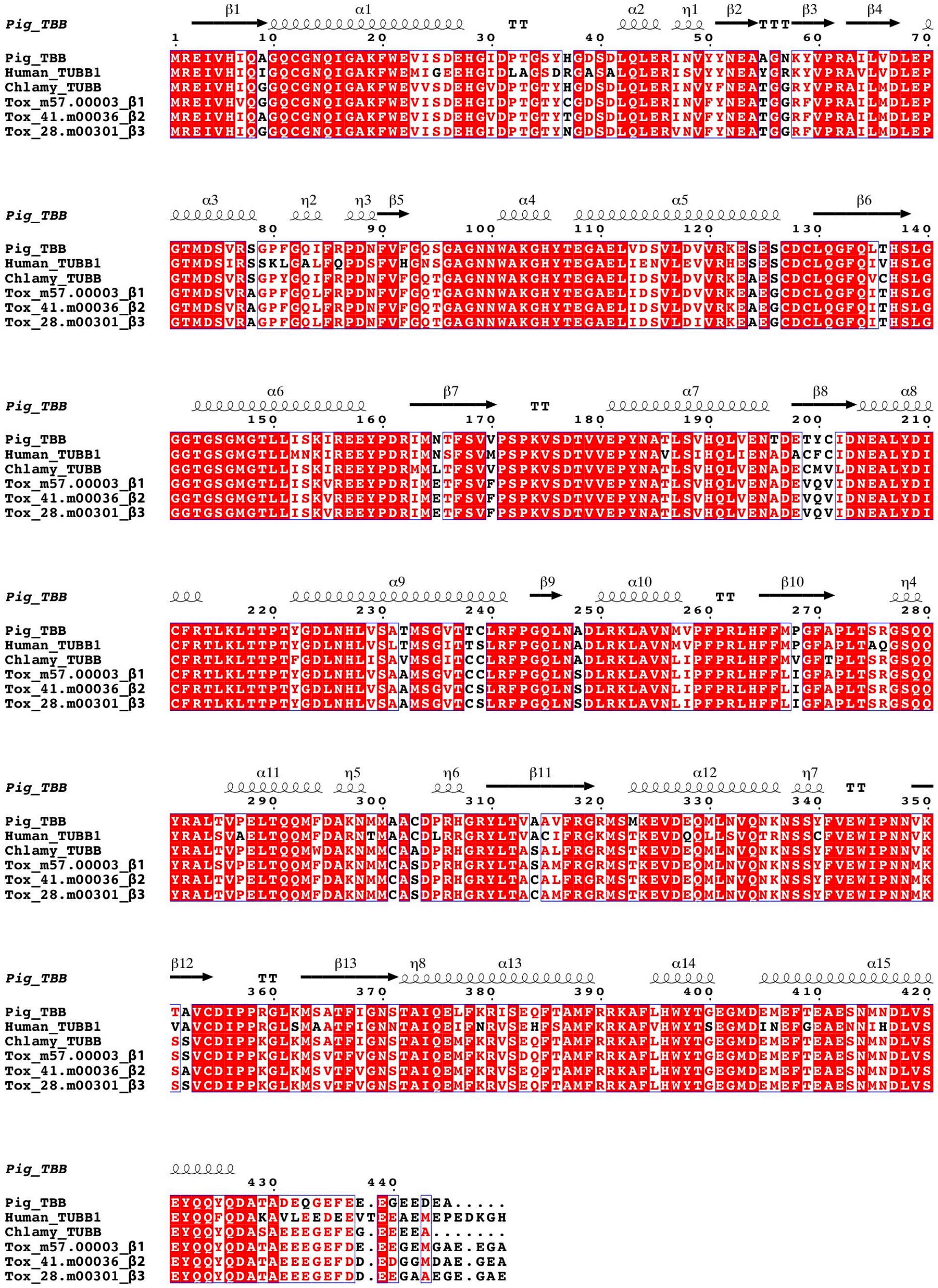

Supplementary Fig. 7 | Sequence alignment of β-tubulin from *T. gondii* and other species. Sequence alignment of β-tubulin from pig, human, *Chlamydomonas* and *T. gondii* (isoforms β1, β2 and β3). Secondary structure elements of β-tubulin from pig are shown above the sequences.

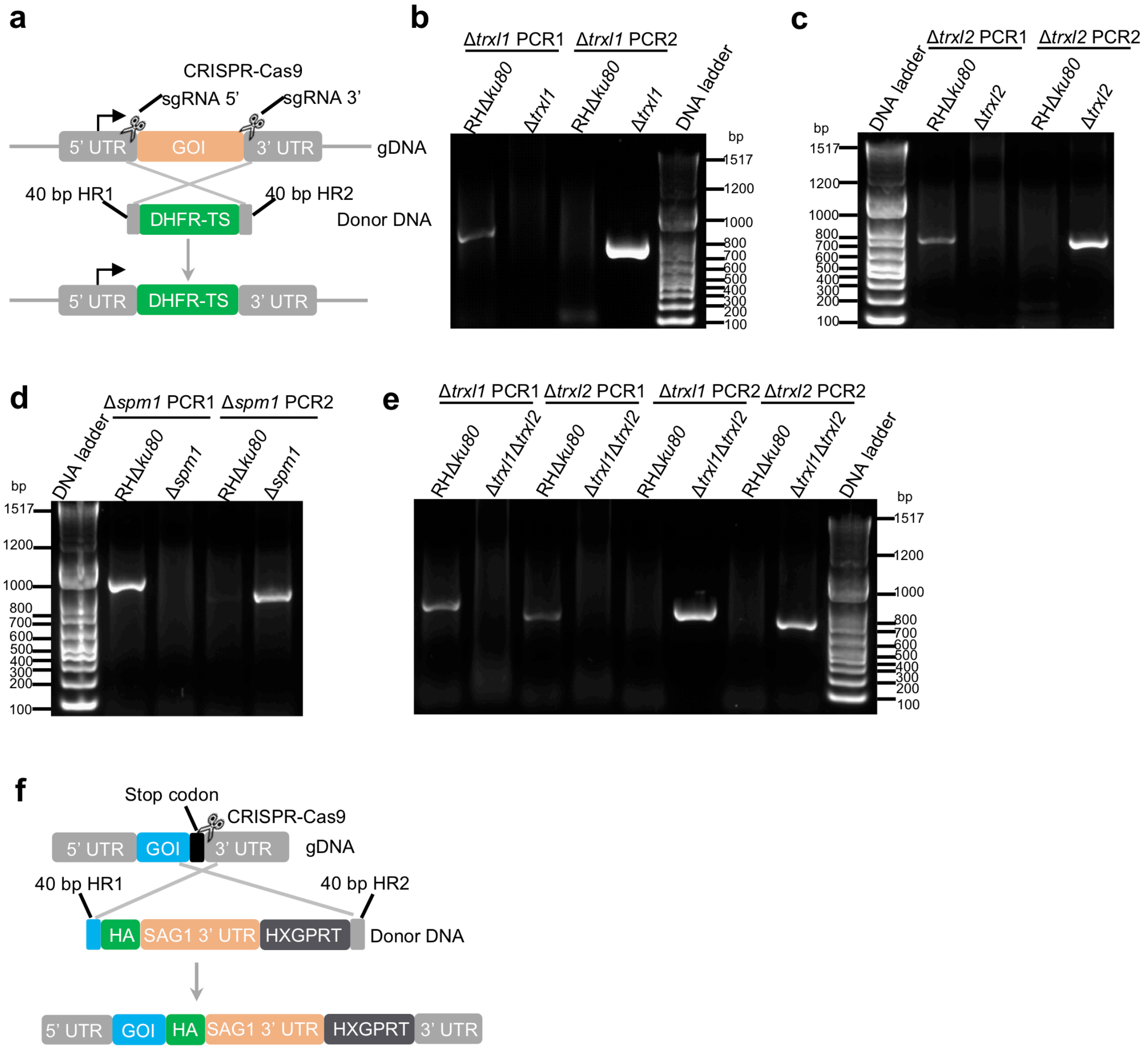

Supplementary Fig. 8 | Generation of *trxl1*, *trxl2* and *spm1* knockouts. **a**, Schematic illustration of genetic knockout for *trxl1*, *trxl2* and *spm1* genes in *T. gondii* using a double CRISPR gRNA system. **b-e**, PCR identification for single knockout strains of Δ*trxl1*, Δ*trxl2,* Δ*spm1* and double knockout strain of Δ*trxl1*Δ*trxl2*. Δ*trxl1* PCR1, Δ*trxl2* PCR1 and Δ*spm1* PCR1 primer sets were used to detect partial fragments of genomic *trxl1*, *trxl2* and *spm1* in those knockouts, respectively; while Δ*trxl1* PCR2 and Δ*trxl2* PCR2 primer sets were used to confirm the 3' integration of DHFR-TS 3' UTR cassette into the gene locus of *trxl1* and *trxl2*, respectively. Δ*spm1* PCR2 primer set was used to probe the 5' integration of DHFR-TS 3' UTR cassette into the gene locus of *spm1*. Experiments in b-e were repeated twice with similar results. **f**, Schematic illustration of CRISPR-Cas9 mediated endogenously C-terminal HA tagging.

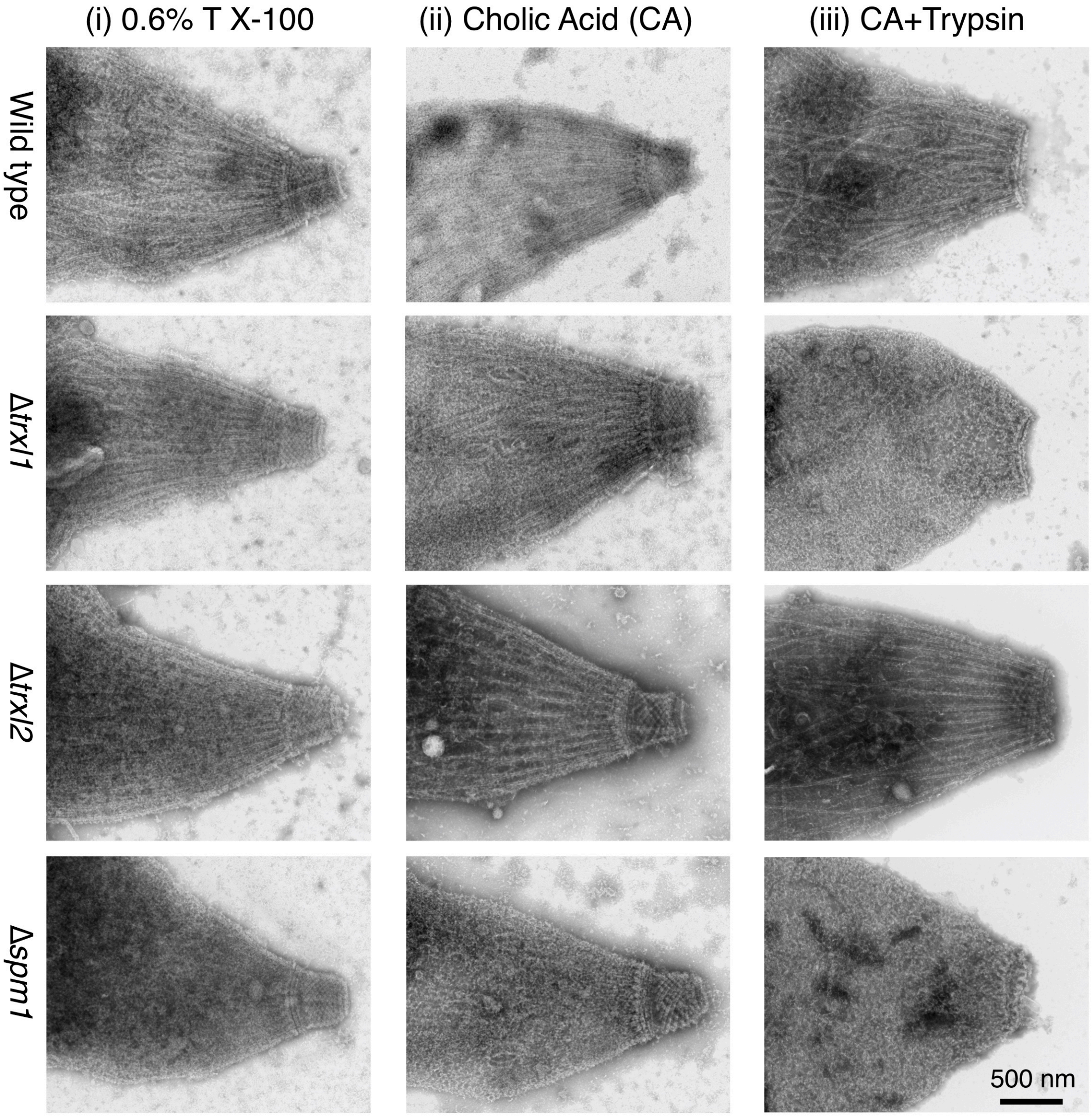

Supplementary Fig. 9 | Close-up view of the apical regions of different *T. gondii* strains upon chemical treatments. Wild-type and three knockout strains (Δ*trxl1*, Δ*trxl2* and Δ*spm1*) of *T. gondii* were challenged by three different chemical treatments: (i) glycerol extraction followed by detergent treatment with 0.6% Triton X-100; (ii) detergent extraction with 1.5% cholic acid; (iii) detergent treatment with 1.5% cholic acid followed by trypsin digestion. The apical regions of each sample were imaged by negative-stain EM at 25,000× magnification. The samples were stained with 1% aqueous phosphotungstic acid (PTA). Ten negative stain images were taken for each condition, and one is shown. In all cases, trypsin digestion leads to loss of the conoids, while the apical polar rings were still present.

Supplementary Table 1. Cryo-EM data collection, refinement and validation statistics

	**EMD-23869, PDB: 7MIZ**
**Data collection and processing**	
Microscope	Titan Krios G3 (Thermo Fisher)
Detector	K2 Summit (Gatan)
Voltage (keV)	300
Nominal magnification	105,000×
Electron exposure (e^-/Å^2)	63.7
Defocus range set during data acquisition (μm)	-1.0 to -3.5
Pixel size (Å)	1.096
Symmetry imposed	C1
Initial particle no. (8 nm)	536,132
Final particle no. (8 nm)	451,204
Map resolution range (Å)	3.3-3.5
**Model composition**	
Chains	100
Residues	27,210
Ligands	26 GTP / 26 GDP / 26 Mg
**Refinement**	
Resolution limit set in refinement (Å)	3.5
Correlation coefficient (CCmask)	0.794
C_{ref} (masked) (Å)	3.45
Root-mean-square deviation (bond lengths) (Å)	0.009
Root-mean-square deviation (bond angles) (Å)	1.053
**Validation**	
MolProbity Score	1.81
Clash score	7.8
Poor rotamers (%)	0.06
Ramachandran (favored) (%)	94.38
Ramachandran (outliers) (%)	0.00

Supplementary Data 1 | Proteins identified in the sample by mass spectrometry. Provided as an Excel table. Two replicates of the treated sample (toxo1 and toxo2) were subject to independent M/S analysis, which yielded similar results. Proteins identified in the cryo-EM structure are highlighted in yellow.

Supplementary Data 2 | All the primers used in this study. Provided as an Excel table.

Supplementary References

69. Robert, X. & Gouet, P. Deciphering key features in protein structures with the new ENDscript server. *Nucleic Acids Research* **42,** W320–4 (2014).
70. Sievers, F. *et al.* Fast, scalable generation of high-quality protein multiple sequence alignments using Clustal Omega. *Mol Syst Biol* **7,** 539 (2011).
