# Supplementary material for: Cryo-EM structure of cortical microtubules from human parasite Toxoplasma gondii identifies their microtubule inner proteins
Source: Nat Commun. 2021 May 24;12:3065. doi: 10.1038/s41467-021-23351-1 (PMC8144581; doi:10.1038/s41467-021-23351-1)
Supplement: Supplementary file 5 — Description of additional supplementary files [file 41467_2021_23351_MOESM5_ESM.docx]

Description of additional supplementary information

Title: supplementary data 1

Description: Mass spectrometry

Title: Supplementary data 1

Description: primers used in this study
